# Argon Induces Protective Effects in Cardiomyocytes during the Second Window of Preconditioning

**DOI:** 10.3390/ijms17071159

**Published:** 2016-07-19

**Authors:** Britta Mayer, Josefin Soppert, Sandra Kraemer, Sabrina Schemmel, Christian Beckers, Christian Bleilevens, Rolf Rossaint, Mark Coburn, Andreas Goetzenich, Christian Stoppe

**Affiliations:** 1 Department of Thoracic & Cardiovascular Surgery, University Hospital RWTH, 52074 Aachen, Germany; Britta Britta.Mayer@rwth-aachen.de (B.M.); jsoppert@ukaachen.de (J.S.); skraemer@ukaachen.de (S.K.); Sabrina.Schemmel@rwth-aachen.de (S.S.); cbeckers@ukaachen.de (C.B.); 2 Department of Anesthesiology, University Hospital RWTH, 52074 Aachen, Germany; cbleilevens@ukaachen.de (C.B.); rrossaint@ukaachen.de (R.R.); mcoburn@ukaachen.de (M.C.); 3 Department of Intensive Care Medicine, University Hospital RWTH, 52074 Aachen, Germany

**Keywords:** argon, late phase of preconditioning, cardioprotection, cardiomyocytes

## Abstract

Increasing evidence indicates that argon has organoprotective properties. So far, the underlying mechanisms remain poorly understood. Therefore, we investigated the effect of argon preconditioning in cardiomyocytes within the first and second window of preconditioning. Primary isolated cardiomyocytes from neonatal rats were subjected to 50% argon for 1 h, and subsequently exposed to a sublethal dosage of hypoxia (<1% O_2_) for 5 h either within the first (0–3 h) or second window (24–48 h) of preconditioning. Subsequently, the cell viability and proliferation was measured. The argon-induced effects were assessed by evaluation of mRNA and protein expression after preconditioning. Argon preconditioning did not show any cardioprotective effects in the early window of preconditioning, whereas it leads to a significant increase of cell viability 24 h after preconditioning compared to untreated cells (*p* = 0.015) independent of proliferation. Argon-preconditioning significantly increased the mRNA expression of heat shock protein (HSP) B1 (HSP27) (*p* = 0.048), superoxide dismutase 2 (SOD2) (*p* = 0.001), vascular endothelial growth factor (VEGF) (*p* < 0.001) and inducible nitric oxide synthase (iNOS) (*p* = 0.001). No difference was found with respect to activation of pro-survival kinases in the early and late window of preconditioning. The findings provide the first evidence of argon-induced effects on the survival of cardiomyocytes during the second window of preconditioning, which may be mediated through the induction of HSP27, SOD2, VEGF and iNOS.

## 1. Introduction

Coronary heart disease is the leading cause of death world-wide, especially in high-income regions [1]. In spite of major improvements in myocardial preservation strategies over the last 20 years, open-heart surgery still remains associated with disconcerting complication rates, with respect to both short- and long-term outcome [2]. However, only a few strategies have been discovered and established that reduce the extent of ischemia/reperfusion (I/R) injury and the resulting outcome of patients. Anesthetic-induced preconditioning (AIP) belongs to the promising approaches, and it exhibits several cardioprotective effects on the vulnerable myocardium during I/R [3,4]. It activates distinct protective signaling pathways within two different time intervals: The classical or first window of preconditioning lasts until 4 h after the preconditioning stimulus, whereas the second window of preconditioning describes a late onset of cardioprotection resulting from de novo synthesis of various proteins [5,6,7,8].

Besides the preconditioning effects of volatile anesthetics, some noble gases are also known to provide organoprotective effects [9,10,11,12]. Xenon shows an early and late onset of protection mediated through the phosphorylation of extracellular signal-regulated kinases (ERK1/2) [13] and protein kinase B (AKT) [10] as well as through the increased synthesis of vascular endothelial growth factor (VEGF) [14,15,16], hypoxia-inducible factor (HIF)-1α [14,15,16], and cyclooxygenase (COX)-2 [17]. Although an increasing body of evidence indicates the neuroprotective and organoprotective properties of argon [9,11,18,19,20,21,22,23], its underlying mechanisms are still partly understood due to the heterogeneity of applied models. Furthermore, the majority of studies reported on argon’s neuroprotective but not organoprotective features in the context of ischemia. Previous studies indicate that argon mediates its neuroprotective effects through activation of ERK1/2, regulation of the Bcl-2 protein family and inhibition of nuclear factor kappa-light-chain-enhancer of activated B cells (NF-κB). In addition, previous studies demonstrated that argon is capable of modulating caspase-3 activity [18,24,25,26,27]. Pagel and coworkers were the first to provide evidence about the preconditioning effects of argon in an in vivo model of myocardial ischemia/reperfusion [21]. Argon has multiple advantages over xenon: it has no anesthetic effect under normobaric conditions [28], no systemic hemodynamic effects [21], and is significantly cheaper than xenon (about eight-fold cheaper in contrast to xenon). These properties may recommend argon as an attractive option for clinical applications, including cardiac surgery.

We hypothesize that argon may provide comparable organoprotective properties as xenon, using similar signaling pathways. Consequently, we aimed to investigate the underlying protective effects and mechanisms of argon preconditioning in an in vitro model of primary isolated cardiomyocytes.

## 2. Results

### 2.1. Cell Survival and Preconditioning Effects

**Figure 1:**
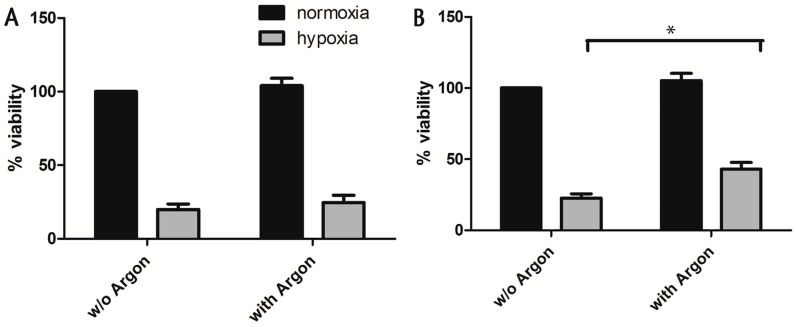

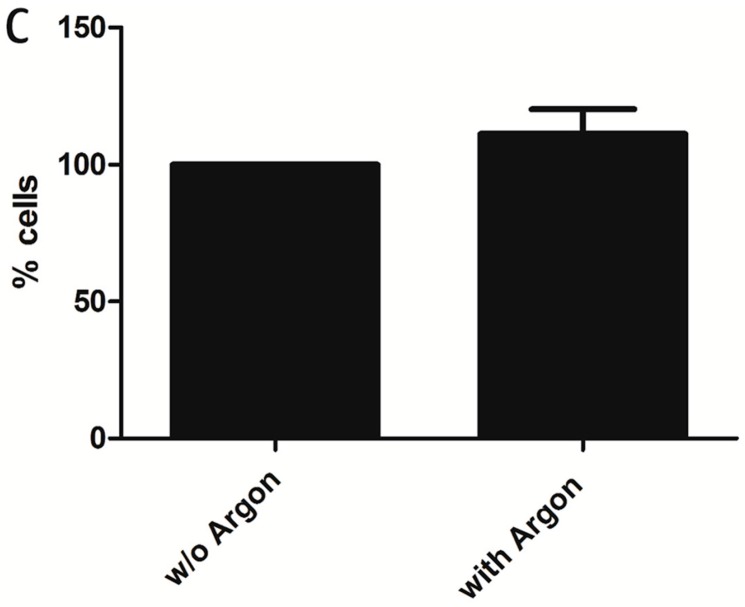
First and second window of preconditioning. Rat cardiac cells (200,000 cells/cm^2^) were subjected either to 50% argon or room air for 1 h. Cells were then subjected to a sublethal dosage of hypoxia (<1% O_2_, 5% CO_2_, 95% N_2_) for 5 h in the first (**A**) and second (**B**) window of preconditioning (directly after preconditioning or 24 h later). Cell survival was assessed via MTT assay 24 h after the ischemic insult and the viability was normalized to normoxic controls; (**C**) Cell proliferation was investigated 24 h after argon treatment using the green fluorescent nucleic acid stain CyQuant. Data represented means ± SEM (standard error of the mean) of three independent experiments for CyQuant and five independent experiments for MTT. *, *p* < 0.05.

To evaluate the preconditioning effect of argon on cell viability, the isolated cells were first subjected to 50% argon (24% N_2_, 21% O_2_ and 5% CO_2_) for 1 h and subsequently exposed to a sublethal dosage of hypoxia (<1% O_2_, 5% CO_2_, 95% N_2_) for 5 h either within the first (0–3 h) or second (24–48 h) window of preconditioning. Within the first window of preconditioning, cell viability did not differ significantly between preconditioned and untreated cells (Figure 1A). In contrast, cell viability was significantly increased in preconditioned cells that were exposed to hypoxia 24 h after preconditioning (second window of preconditioning) in comparison to untreated cells (*p* = 0.015, Figure 1B). In order to rule out that the observed increase of cell viability is due to cell proliferation, the CyQuant assay was performed 24 h after preconditioning. Present data shows that argon treatment did not lead to cell proliferation after 24 h (Figure 1C), which supports our hypothesis that argon itself has preconditioning effects.

**Figure 2:**
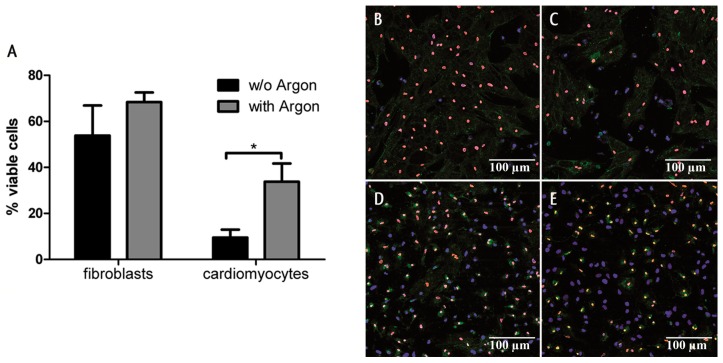
Cell survival in the second window of argon preconditioning (1 h) in either pure cardiomyocytes or fibroblasts. Monocultures of cardiomyocytes and fibroblasts (300,000 cells/ibidi) were treated with 50% argon for 1 h and subjected to 5 h of sublethal hypoxia after 24 h, in the second window of preconditioning. Cells were stained with Hoechst33342 (blue, stains all nuclei), Annexin V (green, stains apoptotic cells), Ethidium homodimer III (red, stains necrotic nuclei). (**A**) Quantification of (**B**–**D**) expressed as % viability (number of necrotic nuclei/all nuclei, normalized to normoxic control cells; (**B**) cardiomyocytes without argon; (**C**) cardiomyocytes with argon; (**D**) fibroblasts without argon; (**E**) fibroblasts with argon. Data represented means ± SEM of five independent experiments. *, *p* < 0.05.

To further confirm these findings, monocultures were stained using the Apoptotic/Necrotic/Healthy Cells Detection Kit. Likewise, the preconditioned cells showed a significantly increased survival compared to cells that were exposed to room air. In addition, we could show in the second window of preconditioning that the protective effects of argon preconditioning were more pronounced in cardiac myocytes than on fibroblasts (Figure 2A–E). 

### 2.2. Induction of Gene Transcription

**Figure 3:**
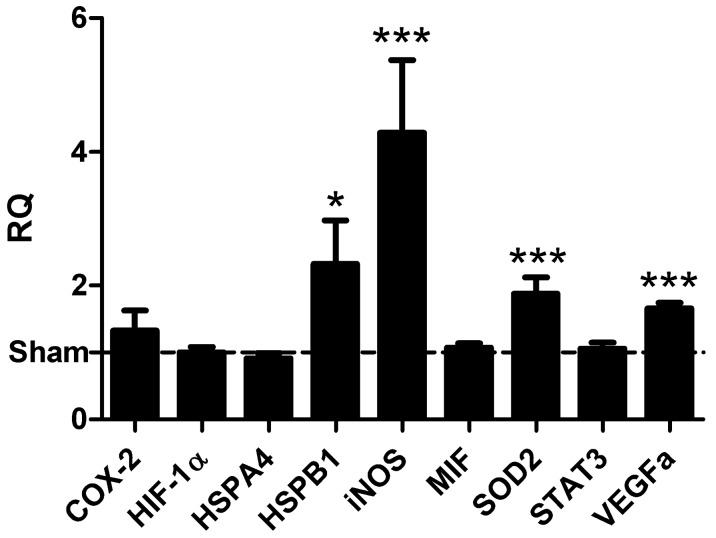
Argon preconditioning induces the transcription of heat shock protein (HSP) B1 (HSP27), superoxide dismutase 2 (SOD2), vascular endothelial growth factor (VEGF), and inducible nitric oxide synthase (iNOS). Eight hours after argon preconditioning, mRNA was isolated, and RT-PCR was performed. mRNA levels were normalized to glyceraldehyde-3-phosphate dehydrogenase (GAPDH) and the relative quantity (RQ) values were calculated according to the ΔΔCt method. Data represented means ± SEM of at least 13 independent experiments. *, *p* < 0.05 and ***, *p* < 0.001.

Since the protective effects within the second window of preconditioning are known to result from de novo synthesis of protective proteins, we measured the mRNA expression of well-known mediators 8 h after preconditioning. As xenon preconditioning was previously shown to induce the production of vascular endothelial growth factor (VEGF) [14,15,16], hypoxia-inducible factor-1α (HIF-1α) [14,15,16], and cyclooxygenase 2 (COX2) [17], we considered these factors in addition to the well-known mediators inducible nitric oxide synthase (iNOS), superoxide dismutase 2 (SOD2), signal transducers and activators of transcription 3 (STAT3), heat shock protein (HSP) B1 (HSP27) and HSPA4 (HSP70) for the analysis after argon preconditioning [29]. Argon preconditioning significantly increased the mRNA expression of HSPB1 (2.32 ± 0.65; *p* = 0.048), SOD2 (1.88 ± 0.24; *p* = 0.001), VEGF (1.66 ± 0.08; *p* < 0.001), and iNOS (4.29 ± 1.09; *p* = 0.001). In contrast, the mRNA expression of COX2 (1.33 ± 0.30; *p* = 0.286), STAT3 (1.06 ± 0.09; *p* = 0.550), HSPA4 (0.91 ± 0.08; *p* = 0.288), and HIF-1α (1.01 ± 0.08; *p* = 0.954) did not change significantly 8 h after preconditioning (Figure 3). 

### 2.3. Protein Expression and Phosphorylation of Kinases

**Figure 4:**
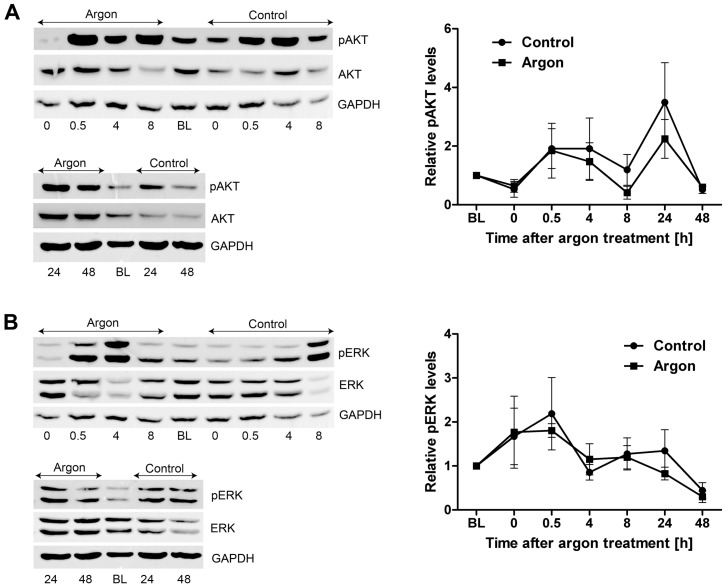
Argon preconditioning does not activate AKT and ERK. Cardiac cells (200,000 cells/cm²) were subjected either to room air (control) or a mixture of argon (argon) for 1 h. Phosphorylation of the protein kinases was assessed at distinct time points (0 h, 0.5 h, 4 h, 8 h, 24 h, and 48 h after preconditioning) by Western blotting and normalized to the unphosphorylated form of the protein. (**A**) Representative immunostaining results as well as the densitometric analysis of AKT; (**B**) Western blot analysis of ERK. Data represented means ± SEM of at least four independent experiments.

Previous studies already showed that, in particular, the activation of pro-survival kinases AKT and ERK1/2 is crucially involved in the protective effects after preconditioning within the first window of preconditioning and they are called classical pro-survival kinases [10,13,18]. In this context, xenon treatment was shown to induce phosphorylation of ERK1/2 between 15 min and 45 min after preconditioning [13] and AKT 15 min after termination of preconditioning [10]. To emphasize these findings, we investigated the effect of argon preconditioning on the activation of ERK1/2 and AKT during the early window of preconditioning. We lysed cells immediately (0 h), 0.5 h, 4 h, 8 h, 24 h and 48 h after argon treatment, and measured the activation of ERK1/2 and AKT via Western blotting. Interestingly, no significant effect of argon was measured on the phosphorylation of ERK1/2 (*p* = 0.671) and AKT (*p* = 0.448, Figure 4) during the entire observation period.

## 3. Discussion

The present study demonstrates a protective effect of argon preconditioning on the survival of cardiomyocytes and fibroblasts that were exposed to a sublethal dose of hypoxia. It appears that according to our data, the protective effects of argon are especially relevant in the second window of preconditioning, which may be mediated through the increased expression of iNOS, SOD2, VEGF and HSPB1 (HSP27). Surprisingly, in the observed time frame, we could not see an effect on gene expression of COX2, HIF-1α, HSP70, and STAT3, which are other well-known mediators of the second window of preconditioning. No protective effects were observed within the first window of preconditioning with respect to cell survival or the involvement of the classical pro-survival kinases AKT and ERK1/2. Both kinases are known to be activated in ischemic-induced preconditioning (IPC), AIP and in preconditioning with the noble gases helium and xenon.

Pharmacological preconditioning belongs to the established preservation strategies of the myocardium that are known to reduce cell apoptosis caused by reperfusion [3,4]. Preconditioning activates distinct pathways, including the pro-survival reperfusion injury salvage kinase (RISK) pathway [30] which inhibits the opening of mitochondrial permeability transition pores (mPTP). Beside the protective effects of volatile anesthetics [3,4], an increasing body of evidence indicates the preconditioning effects of noble gases [31]. While various studies demonstrated the protective effect of xenon treatment [10,12,13,14,15,16,17,32], only little is known about the effects of argon on the injured myocardium and its potential underlying mechanisms. Therefore, we were the first to investigate in an in vitro model the overall effect of argon preconditioning on cardiomyocyte survival. We focused on the underlying mechanisms on a molecular level. Interestingly, we revealed that argon preconditioning significantly increased the survival of cardiac rat cells in the second window of preconditioning, whereas no effect was detected in the first window. Considering the underlying mechanisms, we could demonstrate that argon preconditioning stimulated the expression of SOD2, VEGF, iNOS and HSP27, which belong to the widely established mediators of cardioprotection in the heart [33,34,35]. 

Nitric oxide (NO) is a critical initiator (trigger) as well as effector (mediator) of late ischemic preconditioning [36,37,38]. NO induces angiogenesis and the relaxation of smooth muscle cells (e.g., vasodilatation of coronary arteries) as well as the reduction of oxygen consumption via upregulation of cyclic guanosine monophosphate (cGMP) [39,40]. NO is produced by nitric oxide synthases (NOS) [41]. Guo et al. have found that the targeted disruption of the iNOS gene in mice abrogates delayed cardioprotection, indicating a pivotal role of iNOS as an effector of the late phase of ischemic preconditioning [33,42]. Recent studies indicated that iNOS-mediated cardioprotection is not only a phenomenon seen in IPC but also in anesthetic- and xenon-induced preconditioning [43,44,45]. In accordance to previous findings, we could determine a significant increase in the iNOS gene transcript already 8 h after argon preconditioning. Therefore, we suggest that argon triggers the iNOS expression with subsequent NO production which may contribute to the delayed protection. Nevertheless, further studies are needed to address the contribution of iNOS in argon-induced preconditioning through the use of specific iNOS inhibitors. 

Heat shock proteins represent the first line of defense against acute environmental stress such as heat, ischemia and reperfusion. Several studies demonstrated that increased expression of HSPs may result in enhanced protection against I/R injury through their capacity to remove misfolded proteins, to enhance NO synthesis and to suppress proinflammatory and apoptotic responses by inhibiting the translocation of NF-κB and Bax to mitochondria, respectively [46,47,48,49,50,51]. Thus, HSPs have been suggested as mediators of late preconditioning with special attention to HSP27 (HSPB1). HSP27 is ubiquitously expressed and plays an important role in the reorganization of the cytoskeleton, exhibits chaperone activity [52] and prevents the induction of apoptosis by binding cytochrome c [53]. Previous studies demonstrated that ischemic- as well as xenon-induced preconditioning triggered phosphorylation with subsequent translocation of HSP27 to the actin filament of the cell network via the protein kinase C/p38 mitogen-activated protein kinases/MAP kinase-activated protein kinase 2 (PKC/p38 MAPK/MAPKAPK-2) signal transduction pathway [34,54]. In the current study, an induction of the HSP27 gene transcripts was observed upon argon treatment. We suppose that the argon-mediated increase of HSP27 mRNA may contribute to delayed cardioprotection through enhanced protein folding, degradation of abnormal proteins, inhibition of apoptosis and cytoskeleton stabilization. However, we acknowledge that the influence of chaperone activity based on the phosphorylation status and the location of HSP27 within argon preconditioning needs to be investigated in future studies. 

Mitochondria are the major source of reactive oxygen species (ROS). Detoxification of superoxide by antioxidant enzymes is an important mechanism to protect mitochondria from dysfunction and cell death triggered by oxidative stress—induced lipid peroxidation, DNA damage, protein modifications and opening of mPTPs [55,56]. A possible involvement of mitochondrial manganese superoxide dismutase (SOD2) as an effector of delayed protection was first provided by Hoshida and colleagues, demonstrating that nonlethal hypoxia increases the expression and activity of SOD2 after 24 h [57,58,59,60]. In line with these findings, recent studies investigating the effects of anesthetic- and xenon-induced preconditioning on antioxidant defense mechanisms showed increased activity levels of SOD2 coinciding with cardioprotective effects [61,62]. Our results that argon preconditioning increases the expression of SOD2 already after 8 h support the hypothesis that SOD2 might be a mediator of late cardioprotection induced by argon. The effect of argon preconditioning on the enzymatic activity of SOD2 was not addressed in the present study and warrants further investigation. 

Last, we demonstrated an increased expression of VEGF through argon, which is in accordance with a study from Goetzenich et al. showing an augmented VEGF mRNA content upon xenon treatment [43]. VEGF is known to induce angiogenesis and thus may enhance the reperfusion and oxygen supplementation in the heart at a later stage. Although the transcription is known to be mediated by HIF-1α [63], no significant change was observed for the expression of HIF-1α 8 h after preconditioning, indicating a non-classical activation of this factor. Nevertheless, it remains unknown whether HIF-1α increased at a different time point, which was not considered in this study. In this context, Rizvi and co-workers showed a significant decrease of HIF-1α between 16 and 24 h after preconditioning [16,64]. Thus, HIF-1α might already increase earlier (e.g., <8 h after preconditioning) to activate the transcription of VEGF, before it again decreases to normal levels. Furthermore, it is known that the transcription of VEGF may be mediated via an alternative pathway, for example through secretion of different growth factors and cytokines [65,66].

**Figure 5:**
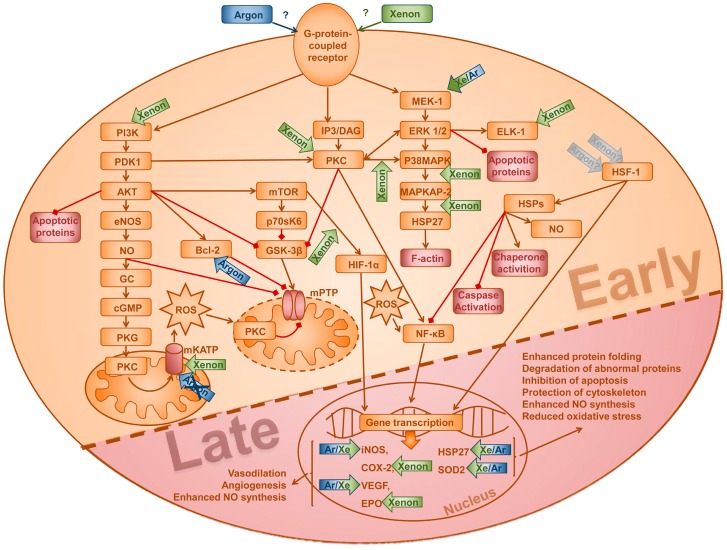
Supposed model of underlying mechanisms of protective effects of early and delayed preconditioning mediated by xenon and argon. This figure is a summary of previous studies that investigated the mechanisms of xenon and argon that may contribute to protection in the first and second window of preconditioning. Please note: this scheme does not comprise information about species, model (in vitro or in vivo), cell type, concentration or duration of argon or xenon. The plotted arrows for xenon (green) and argon (blue) indicate their up-regulatory properties on different kinases and molecules. Brown arrow-headed lines denote an activating effect, whereas red square-headed lines indicate a suppressive effect. PI3K, phosphoinositide 3-kinase; PDK1, phosphoinositide-dependent kinase-1; Bcl-2, B-cell lymphoma 2; eNOS, endothelial nitric oxide synthase; iNOS, inducible nitric oxide synthase; NO, nitric oxide; mTOR, mechanistic target of rapamycin; IP3, inositol trisphosphate; DAG, diacylglycerol; PKC-ε, protein kinase C epsilon; GSK-3β, glycogen synthase kinase-3 beta; MEK-1, mitogen-activated protein kinase kinase 1; ERK1/2, extracellular signal-regulated protein kinases 1/2; p38 MAPK, p38 mitogen-activated protein kinases; MAPKAP-2, MAP kinase-activated protein kinase 2; HSP, heat shock protein; mKATP, mitochondrial potassium ATP channels; mPTP, mitochondrial permeability transition pore ; HIF-1α, hypoxia-inducible factor-1 alpha; HSF-1, heat shock factor-1; Elk-1, ETS domain-containing protein; COX-2, Cyclooxygenase-2; EPO, erythropoietin; VEGF, vascular endothelial growth factor.

We did not reveal any effect on the classical pro-survival kinases ERK1/2 and AKT, which are known to be involved in the underlying mechanisms of classical preconditioning. However, our findings that ERK1/2 and AKT are not phosphorylated upon argon treatment are in line with the lack of protective effects within the first window of preconditioning. In contrast, Pagel and coworkers demonstrated an argon-induced reduction of infarct size during the first window of preconditioning in rabbits [21]. This observed discrepancy between Pagel’s and our study could be due to remarkable differences in the applied protocol including type of model (in vivo vs. in vitro), species (rabbit vs. rat), argon concentration (70% vs. 50%) and type of argon application (cyclic vs. one time). Besides, Pagel et al. did not investigate the contribution of the RISK pathway upon argon preconditioning or the role of argon in the second window of preconditioning [21]. While extensive data exists concerning the signaling of AIP, data on noble gases is still scarce. Figure 5 depicts the known pathways involved in the development of early and late signaling, emphasizing the contributors that are known to be also mediated by the noble gases argon and xenon. 

### Limitations

We acknowledge that our study has several limitations. First, the results of the studies have to be considered as purely hypothesis-generating, data have been compared to a control group and need further confirmation in following in vivo models. Nevertheless, it has to be acknowledged that distinct signaling process can only be investigated in separated in vitro models. Second, we have to concede that the concentration of 50% argon was tested for one specific time interval, which is in accordance with the study of Loetscher and co-workers [20] that showed the best neuroprotective effect at the same argon concentration of 50% in a stroke model. Indeed, systematically, studies that address a dose-dependent effect of argon are still lacking and thus further studies are needed to focus on the ideal argon concentration and timing of application. However, it remains speculative if a better cardioprotective effect may be achieved using a different argon concentration and/or duration of application. Third, we have only considered the activation of protective enzymes, transcription of genes and outcome measurements at distinct time points. Thus, we cannot rule out if an activation/deactivation of enzymes occurs at different time points, which were not investigated as part of this study. However, all time points were within the well-known windows of early and late preconditioning, so that we assume to provide the major characteristics after argon preconditioning in a time-dependent manner. Further studies are needed—in extension to this analysis—to address the exact kinetics of the involved mechanisms.

## 4. Materials and Methods

### 4.1. Ethics Approval

All experiments were performed in accordance with the local institution’s Ethical Review Committee and were approved by an animal protection representative at the Institute of Animal Research of the RWTH Aachen University Hospital in accordance with German Animal Protection Law §4, Section 3. All experimental procedures were approved by the Animal Care and Use Committee of the local authorities (AZ 50.203.2 AC, LANUV NRW, Essen, Germany).

### 4.2. Reagents

All reagents were obtained from Sigma (St. Louis, MO, USA), Merck (Darmstadt, Germany) and Roth (Karlsruhe, Germany). Antibodies were obtained from Cell Signaling Technologies (Cambridge, UK) and diluted in Tris-buffered saline (TBS) containing 5% bovine serum albumin (BSA) and 0.05% Tween 20, unless stated otherwise. 

### 4.3. Cell Culture

In an established model [43,67], cardiac ventricular heart cells were isolated from zero to four-day-old Wistar rat pups by trypsin and DNase digestion as previously described in order to analyze the effect of argon on a molecular level [67]. Briefly, ventricles were put into ice-cold calcium and bicarbonate free Hanks buffer (CBFHH buffer) with 20 mM 4-(2-hydroxyethyl)-1-piperazineethanesulfonic acid (HEPES), pH 7.4, 140 mM NaCl, 5.4 mM KCl, 0.81 mM MgSO_4_, 0.44 mM KH_2_PO_4_, 0.34 mM Na_2_HPO_4_, and 5.6 mM glucose), minced into pieces <1 mm and incubated in several cycles first with 0.25% trypsin (Life Technologies, New York, NY, USA) and then with DNAse (Life Technologies). The first emerging supernatant was discarded, the following supernatants containing vital isolated heart cells were collected and centrifuged (15 min at 60× *g* at 4 °C). The resulting cell pellet was resuspended in dulbecco’s modified eagle medium (DMEM) containing 2 mM l-glutamine, 10% horse serum, 2% chicken embryo extract and 100 U/mL penicillin/streptomycin and filtered (100 µm filter BD Falcon, Durham, NC, USA). To mimic the in vivo situation in the heart, which consists of one third of cardiomyocytes and two third of fibroblasts, cells were plated at 200,000 cells/cm^2^ on cell culture dishes coated with 5 mg/mL fibronectin in 0.02% gelatin (mixed culture). To create a pure cell culture containing either cardiomyocytes or fibroblasts, cells were layered over Percoll (GE Healthcare, Little Chalfont, UK) and centrifuged (30 min, 1000× *g*, 4 °C). The cardiomyocytes are located at the interface between Percoll and medium, whereas the fibroblasts were located in the medium above the interface [36,68]. The separated cells were plated at 200,000 cells/cm^2^ on cell culture dishes coated with 5 mg/mL fibronectin in 0.02% gelatin, and cultivated in medium for 10 to 14 days in order to develop an adult phenotype [69]. A representative picture of all three cell cultures is given in Figure S1, confirming the purity of the used cell cultures. 

### 4.4. Experimental Set-up and Preconditioning

**Figure 6:**
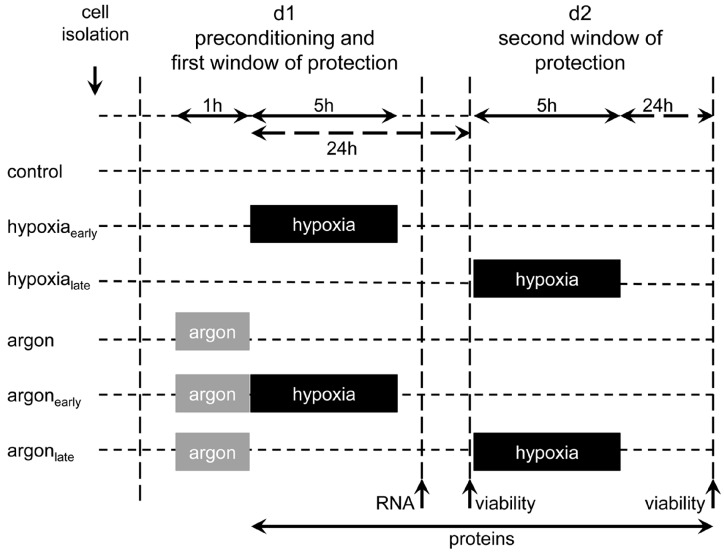
Experimental set-up. Isolated rat heart cells, pure cardiomyocytes, and fibroblasts were subjected either to 50% argon (50% Ar, 24% N_2_, 21% O_2_, 5% CO_2_) or room air (74% N_2_, 21% O_2_, 5% CO_2_) for 1 h at 37 °C. Cells were either challenged within the first (0–3 h) or second window (24–48 h) of preconditioning with an ischemic insult of 5 h. Cell survival was assessed 24 h after prolonged hypoxia via MTT assay. To unravel the underlying mechanisms, mRNA expression was analyzed 8 h after argon treatment. For investigating the activation of pro-survival kinases, cell lysates were prepared at different time points (0 h, 0.5 h, 4 h, 8 h, 24 h, 48 h) after preconditioning.

The experimental set-up is illustrated in Figure 6. The isolated cells were randomized into six groups: control and normoxia (control), control with an early (hypoxia_early_) and late onset of hypoxia (hypoxia_late_), argon and normoxia (argon) and two groups regarding early (argon and early hypoxia, argon_early_) and late preconditioning (argon and late hypoxia, argon_late_). Within each group, all experiments were carried out on individual cell preparations.

For preconditioning, the cells were exposed for one hour to a gas mixture of argon (50% Ar, 24% N_2_, 21% O_2_ and 5% CO_2_), which is in accordance to previous encouraging experiments [20]. Immediately or 24 h after preconditioning, a sustained period of hypoxia (<1% O_2_, 5% CO_2_, 95% N_2_) was performed for 5 h in a self-designed heating chamber (WALLA GmbH, Würselen, Germany) under low flow conditions. The chamber was connected to a multichannel flow regulator (Bronkhorst-Mättig, Kamen, Germany) and the gas concentration was monitored using a Datex Ohmeda multichannel patient monitor (GE Healthcare, Munich, Germany) [43]. The control group was maintained throughout the experiment in a humidified incubator at 37 °C (74% N_2_, 21% O_2_, 5% CO_2_). All subsequent analyses were performed by a blinded researcher.

### 4.5. Cell Viability after Terminal Hypoxia

Cell viability was measured 24 h after I/R using two different methods: The MTT assay, as a quantitative method, was performed as described previously [70]. Briefly, the cells were incubated with 5 mg/mL MTT (3-(4,5-dimethylthiazol-2-yl)-2,5-diphenyltetrazolium bromide) for 10 min, which is metabolized by viable cells to a blue formazan derivative. Subsequently, the cells were lysed in 300 µL 0.04 M HCl in isopropanol and diluted with 300 µL destilled water. The absorbance was measured in a Tecan infinite M200 ELISA reader (Tecan, Männedorf, Switzerland) at 570 nm. 

The Apoptotic/Necrotic/Healthy Cells Detection Kit (PromoKine, Heidelberg, Germany), a qualitative assay to analyze cell viability, was used to stain apoptotic and necrotic cells [43]: 200,000 to 300,000 cardiomyocytes were plated in 35 mm microdishes (Ibidi, Martinsried, Germany) for 10 to 14 days and the same number of fibroblasts were plated for three to four days. The cells were washed with PBS and incubated with hoechst33342, annexin V and ethidium homodimer III according to manufacturer’s instructions. Cells were visualized by laser scanning fluorescence microscopy using an LSM710 confocal microscope (Zeiss, Jena, Germany).

### 4.6. Proliferation Assay

Cell proliferation was measured using the green fluorescent nucleic acid stain (CyQuant, Life Technologies) [71]. After exposure to argon, the cells were frozen for 10 min at −80 °C and incubated with the assay solution containing the CyQuant dye according to the instructions for 10 min. Chemiluminescence was measured at 480 nm/520 nm using the Tecan infinite M200 ELISA reader.

### 4.7. Western Blotting

At different time points after preconditioning, the cells were lysed with radioimmunoprecipitation assay (RIPA) buffer (0.15 M NaCl, 1% NP40 (*v*/*v*), 0.02 M sodium deoxycholate, 0.04 M Tris HCl), containing phosphatase inhibitor cocktail (PhosSTOP Roche, Mannheim, Germany) and protease inhibitor cocktail (complete, Roche, Mannheim, Germany), on ice for 10 min and subsequently centrifuged in a QIA Shredder column (QIAGEN, Hilden, Germany) to shear genomic DNA. Protein concentration was measured using a colorimetric assay (DC Protein Assay, Bio-Rad, München, Germany). 15 µg protein were mixed with Laemmli sample buffer, heated to 95 °C and subjected to SDS-PAGE and semi-dry Western blotting. For detection, the membranes were probed with anti-pAKT (CS4060 1:2000), anti-pERK (CS4370 1:2000) and anti-GAPDH (CS5174 1:1000) over night. Blots were developed with Clarity Western ECL substrate (BioRad, München, Germany) using a peroxidase-conjugated anti-rabbit antibody (CS7074 1:1000) which was incubated for 2 h. The resulting chemiluminescence was detected using the ChemiDoc MP System (BioRad, München, Germany). Band intensities were analyzed using Image Lab software (BioRad, München, Germany), and normalized to unphosphorylated protein. For reprobing, membranes were stripped (62.5 mM Tris HCl, 2% SDS, 400 µL 2-mercaptoethanol) for 20 min at 55 °C and after blocking with 5% BSA incubated with anti-AKT (CS4691 1:1000) and anti-ERK (CS4695 1:1000) over night. 

### 4.8. Quantitative Real Time Polymerase Chain Reaction

Total RNA was isolated 8 h after preconditioning using the Nucleo Spin RNA/Protein kit (Machery-Nagel, Düren, Germany) and reverse-transcribed into cDNA using a high-capacity cDNA reverse transcription kit (Applied Biosystems, Carlsbad, CA, USA). The PCR was performed using 50 ng cDNA and TaqMan probes (TaqMan universal PCRmix, Applied Biosystems; COX2 Rn01483828_m1, GAPDH Rn99999916_s1, HIF-1α Rn00577560_m1, HSPA4 Rn00596544_m1, HSPB1 Rn00583001_g1, iNOS Rn00561646_m1, SOD2 Rn00690587_g1, MIF Rn00821234_g1, STAT3 Rn00562562_m1, VEGF Rn01511601_m1) on a StepOne Plus Real-Time PCR Systems (Applied Biosystems, Carlsbad, CA, USA). The relative quantity (RQ) values were calculated according to the ΔΔ*C*_t_ method.

### 4.9. Statistical Analysis

The results are presented as mean ± SEM unless stated otherwise [72]. Data were analyzed by one-way-ANOVA and post-hoc Bonferroni’s multiple comparison tests with GraphPad Prism 5 software (GraphPad Software Inc., San Diego, CA, USA) to compare differences between multiple groups and Student’s unpaired t-test and one sample *t*-test (PCR data) when analyzing two groups. A value of *p* < 0.05 was considered to be statistically significant.

## 5. Conclusions

In conclusion, this study extends the increasing evidence of argon’s protective effects on the survival of cardiomyocytes during the second window of preconditioning, whereas no effect was observed in the first window. The observed conditioning effects may result from the significantly increased synthesis of SOD2, iNOS, VEGF and HSP27, which are well-known mediators of the late phase of protection induced by preconditioning with nonlethal hypoxia or xenon. Argon seems to share joint features with xenon, especially concerning organoprotective effects in the second window of preconditioning and the up-regulation of important mediators. However, considerable differences seem to exist in the signaling pathway. Additional studies are needed to validate these findings in in vivo models.

## Supplementary Materials

Supplementary materials can be found at http://www.mdpi.com/1422-0067/17/7/1159/s1.
