# Supplementary material for: Argon Induces Protective Effects in Cardiomyocytes during the Second Window of Preconditioning
Source: Int J Mol Sci. 2016 Jul 19;17(7):1159. doi: 10.3390/ijms17071159 (PMC4964531; doi:10.3390/ijms17071159)
Supplement: Supplementary file 1 [file ijms-17-01159-s001.pdf]

Supplementary Materials: Argon Induces Protective Effects in Cardiomyocytes during the Second Window of Preconditioning

Britta Mayer, Josefin Soppert, Sandra Kraemer, Sabrina Schemmel, Christian Beckers, Christian Bleilevens, Rolf Rossaint, Mark Coburn, Andreas Goetzenich and Christian Stoppe

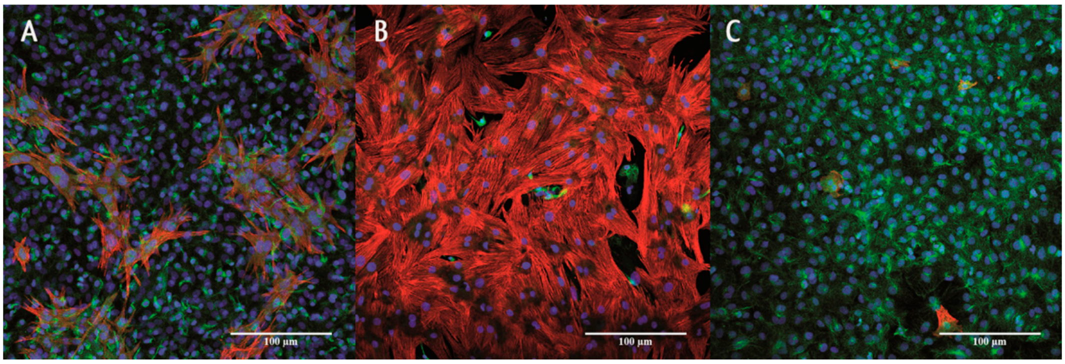

Figure S1. Purity of cell cultures. Cardiac cells were isolated from neonatal rats by trypsin digestion. Further separation of cardiomyocytes and fibroblasts was achieved by percoll density gradient centrifugation. Cells were stained with the cardiomyocyte marker Troponin T (red) and a fibroblast marker Vimentin (green). Nuclei were labelled with Dapi (blue). (**A**) Mixed culture of fibroblasts and cardiomyocytes; (**B**) Monoculture of cardiomyocytes; (**C**) Monoculture of fibroblasts.
